# Virulence of *Mycobacterium tuberculosis* after Acquisition of Isoniazid Resistance: Individual Nature of *katG* Mutants and the Possible Role of AhpC

**DOI:** 10.1371/journal.pone.0166807

**Published:** 2016-11-28

**Authors:** Luisa Maria Nieto R, Carolina Mehaffy, Elizabeth Creissen, JoLynn Troudt, Amber Troy, Helle Bielefeldt-Ohmann, Marcos Burgos, Angelo Izzo, Karen M. Dobos

**Affiliations:** 1 Department of Microbiology, Immunology and Pathology, Colorado State University, Fort Collins, Colorado, United States of America; 2 School of Veterinary Science, University of Queensland, Gatton, Queensland, Australia; Australian Infectious Diseases Research Centre, University of Queensland, St Lucia, Queensland, Australia; 3 School of Chemistry and Molecular Biosciences, University of Queensland, St Lucia, Queensland, Australia; 4 Division of Infectious Diseases, Department of Medicine, University of New Mexico, Albuquerque, New Mexico, United States of America; Johns Hopkins University, UNITED STATES

## Abstract

In the last decade, there were 10 million new tuberculosis cases per year globally. Around 9.5% of these cases were caused by isoniazid resistant (INHr) *Mycobacterium tuberculosis (Mtb)* strains. Although isoniazid resistance in *Mtb* is multigenic, mutations in the catalase-peroxidase (*katG*) gene predominate among the INHr strains. The effect of these drug-resistance-conferring mutations on *Mtb* fitness and virulence is variable. Here, we assessed differences in bacterial growth, immune response and pathology induced by *Mtb* strains harboring mutations at the N-terminus of the *katG* gene. We studied one laboratory and one clinically isolated *Mtb* clonal pair from different genetic lineages. The INHr strain in each pair had one and two *katG* mutations with significantly reduced levels of the enzyme and peroxidase activity. Both strains share the V1A mutation, while the double mutant clinical INHr had also the novel E3V *katG* mutation. Four groups of C57BL/6 mice were infected with one of the *Mtb* strains previously described. We observed a strong reduction in virulence (reduced bacterial growth), lower induction of proinflammatory cytokines and significantly reduced pathology scores in mice infected with the clinical INHr strain compared to the infection caused by its INHs progenitor strain. On the other hand, there was a subtle reduction of bacteria growth without differences in the pathology scores in mice infected with the laboratory INHr strain. Our results also showed distinct alkyl-hydroperoxidase C (AhpC) levels in the *katG* mutant strains, which could explain the difference in the virulence profile observed. The difference in the AhpC levels between clonal strains was not related to a genetic defect in the gene or its promoter. Cumulatively, our results indicate that the virulence, pathology and fitness of INHr strains could be negatively affected by multiple mutations in *katG*, lack of the peroxidase activity and reduced AhpC levels.

## Introduction

Tuberculosis (TB) has killed more people than any other infectious disease in history. In the last century, the number of deaths due to TB has declined due to the discovery of anti-TB chemotherapy, but HIV coinfection as well as the increasing number of drug resistant cases has worsened the global burden of the disease [1]. Isoniazid (INH) is one of the most effective drugs within a multi-drug regimen to treat patients with active TB. INH is also often used as monotherapy for latent TB infection (LTBI), and in some countries as a preventive therapy for people living with HIV [2]. According to the World Health Organization, around 9.5% of the total global TB cases were INH resistant in the last decade [3]. INH is a prodrug that requires oxidative activation by the bacterial catalase-peroxidase enzyme (KatG), forming INH-NAD adducts [4]. Mutations in *katG* gene are the most common cause of INHr TB cases [5–9], however, mutations in more than 20 genes have been identified in INH resistant (INHr) *Mtb* strains and are listed in the TB Drug Resistance Mutation Database as potential causes for INHr (https://tbdreamdb.ki.se/Data/DrugArea.aspx?AreaID=INH) [8, 10]. These genes are involved in drug acetylation (*nat*), efflux (*efpA*) and mycolic acid synthesis (*inhA*, *kasA*) among other bacterial mechanisms. The effect of these mutations on *Mtb* fitness and virulence is variable. The most frequent *katG* mutation found in multidrug resistant TB cases (Ser315Thr) is associated with minimal to no reduction in fitness [11, 12], with a functional catalase-peroxidase activity [12]. Conversely, total deletion and other mutations in the *katG* gene result in severely attenuated *Mtb* strains [12–14].

In general, some *katG* mutations render *Mtb* strains sensitive to endogenous or exogenous peroxides, generated during bacteria respiration or by phagocytes during infection respectively [15]. This may be the result of mutations that besides preventing the activation of INH, also affect the peroxidase domain of KatG [16]. Previous studies have indicated the role of the alkyl hydroperoxidase C (AhpC) as an important compensatory mechanism in INHr strains. The last because KatG and AhpC share organic peroxides and reactive nitrogen intermediates as substrates [17, 18]. For instance, Sherman *et al*., demonstrated that *katG* mutant clinical isolates had a wide range of increased levels of the AhpC compared with the reference strain H37Rv. These *katG* mutant strains with high AhpC levels had at least one mutation in the promoter region of the *ahpC* gene [18]. Although the levels of AhpC in some *Mtb* clinical isolates have been almost imperceptible, the inhibition of AhpC in INHr strains affect their ability to react against reactive oxygen and nitrogen intermediates delivered by the host immune response [17]. Thus, AhpC is a relevant enzyme for select *Mtb* INHr strains to maintain bacterial virulence.

*Mtb* virulence is associated with both the lack of an effective immune response in the host and ability of the bacteria to invade, survive and multiply in the host [19]. After *Mtb* interacts with macrophages and dendritic cells from the human or murine host, cytokines of the T helper type 1 (Th1) response, such as tumor necrosis factor (TNF)-α and interferon-γ (IFNγ), are crucial for the control of *Mtb* infection [20]. However, a “dynamic balance” between all Th cell subsets is required to avoid an exacerbated inflammatory response in the lung tissue and to accomplish a successful bacterial inhibitory response [21, 22]. *Mtb*-host interaction and bacterial fitness are also variable in both drug susceptible and resistant isolates depending on the *Mtb* genetic background [23, 24]. Additionally, genetic diversity in the same host can be due to two mechanisms: clonal strains that are derived due to “within-host mutations” from a single progenitor strain or the presence of mixed infections with polyclonal strains [25].

In this study, we present the results of the analysis of clonal strains that were obtained in the clinical and laboratory setting. The mutations generated in the clonal strains are likely to be caused by the exposure of anti-TB therapy that regularly leads to acquired drug resistance events [25]. Therefore, clonal strains are an excellent strategy to study the effect of drug-resistance-conferring mutations derived from a real clinical setting. Our aim was to analyze the virulence profile of the INHr strains due to mutations in the *katG* gene with a description of their *in vitro* KatG levels, peroxidase activity, and the possible compensatory levels of the AhpC enzyme. Virulence and fitness were measured looking at bacterial growth, immune response and pathology induced by the clonal pairs of *Mtb* strains in the mouse model of TB infection.

## Materials and Methods

### Ethics statement

All experiments involving mice were approved by Colorado State University’s Institutional Animal Care and Use Committee-IACUC (protocol #13-4509A). Samples obtained from the human subject were obtained from culture positive *Mtb* patients in a study of TB transmission in San Francisco following approval from Stanford University and the University of California San Francisco’s Institutional Review Boards [13, 26].

### Bacterial strains

Clinical and laboratory strains of *Mtb* were cultured in Proskauer-Beck (PB) media to prepare the infectivity stocks as previously described [27]. Bacterial stocks were stored in PB media with 20% glycerol at -80°C until used for the mouse infection study. The clinical strains belong to the T genotype of *Mtb* (previously determined by spoligotyping [28] and RFLP-*IS*6110 [29]) and were isolated from a pulmonary TB, smear positive, HIV negative patient from San Francisco, U.S.A. The patient was cured and did not reactivate the disease in a two-year follow up. The first strain isolated was susceptible to all drugs tested while the second one was resistant to INH only. Both strains were provided to Colorado State University (to the laboratory of Dr. Ian Orme, with subsequent provision to the laboratory of Dr. Karen Dobos) by Dr. Marcos Burgos. The laboratory strain H37Rv (Trudeau Mycobacteria Culture Collection # 107) was part of our own bacterial collection provided to Johns Hopkins University. The mutant pair was provided as a kind donation from Doctors Gyanu Lamichhane and Eric Nuermberger at the Center of Tuberculosis Research of Johns Hopkins University (Baltimore, MD). This laboratory INHr strain was obtained from a mouse initially infected with the H37Rv strain that developed the INHr phenotype after treatment with INH [30].

Mutation analysis in the *katG* gene previously done by PCR-sequencing [31] confirmed the mutations Val1Ala and Glu3Val for the clinical INHr strain and Val1Ala only for the laboratory INHr strain [30]. The Val1Ala mutation has been previously identified in clinical INHr TB cases [32, 33], while the Glu3Val is a novel mutation. Prior to infecting animals, the growth of each strain was assessed in BACTEC media using the MGIT^™^320.

### KatG levels by western blot and peroxidase activity testing

Three biological replicates of each strain were cultured in 1 L of glycerol alanine salt (GAS) media for three weeks in constant agitation at 37°C. After this time, culture supernatants and cytosolic fractions from the cells were purified as previously described [34]. Protein concentration and buffer exchange from the cytosol and culture filtrate proteins (CFP) preparations were accomplished using a 3,000 KDa Amicon^®^ Ultrafiltration unit. Quantification of total protein was determined by the bicinchoninic acid (BCA) method (Thermo Scientific Pierce).

For western blot analysis, 5 μg of total protein of cytosol and CFP of two biological replicates for each strain were transferred into a nitrocellulose membrane. Anti-*Mtb* KatG (clone IT-57) mouse monoclonal antibody was used as the primary antibody at a 1:1,000 dilution (available through Biodefense and Emerging Infections Research Resources Repository-BEI, https://www.beiresources.org/), the secondary antibody goat anti-mouse IgG (H+L)-HRP conjugated (Thermo Scientific) was used with the chemiluminescent detection system (SuperSignal West Pico Stable peroxide solution, Thermo Scientific Pierce) to detect KatG. Recombinant KatG protein (also from BEI) was used as positive control. Western blot images were taken with the Chemi-Doc XRS and the Image Lab software version 3.0 (BioRad).

For the peroxidase activity assay, total protein of each cytosol and CFP samples were diluted to 1.6 μg/μl with 1X PBS buffer. An end point assay was performed to evaluate the peroxidase activity at 25°C by adding 3,3′,5,5′-Tetramethylbenzidine liquid substrate system (TMB, Sigma-Aldrich). The TMB reagent (that already contains hydrogen peroxide) was added to the sample in a 1:1 volume ratio (final volume: 80 μL). After 15 minutes incubation, the reaction was stopped by adding 40 μL of 0.5 M H_2_SO_4_ solution and then the optical density (OD) was read at 450 nm [35]. A standard curve was prepared by making five serial dilutions of recombinant KatG within a concentration range from 0.125 mg/mL to 2 mg/mL, to infer the peroxidase activity of the soluble fractions of the *Mtb* cultures.

### Analysis of the alkyl hydroperoxidase C (AhpC)

A PCR-Sanger sequencing analysis of the *ahpC* gene (588 bp) and the *oxyR-ahpC* intergenic region (105 bp) (where the *ahpC* promoter is located) was performed to determine the presence of possible compensatory SNPs in the INHr strains. For the amplification of the DNA sequence of interest, primers described in S1 Table were used, following the reaction and cycling conditions previously described by Datta *et al* [31]. Sequencing analyses were performed by the company Genewitz^®^ (New Jersey, U.S.A.). Sequence alignment analyses were done using the Vector NTI Express version 1.4.0. Additionally, the levels of the AhpC protein in the soluble fractions (5 μg of total protein of cytosol and CFP) from the bacterial cultures were assessed through western blot. Anti-*Mtb* AhpC rabbit polyclonal was used as the primary antibody at a 1:6,000 dilution. This antibody was kindly provided by Doctors Kristin Burns-Huang and Carl Nathan from Weill Cornell Medical College (New York, NY) [17]. The secondary antibody was a goat Anti-Rabbit F(ab)2 fragment (Thermo Scientific) and the subsequent detection step was done as previously described for the KatG western blot.

### Mouse infection

Groups of C57BL/6 female mice (n = 35/grp), 6–8 weeks old (The Jackson Laboratory, Bar Harbor, ME), were infected with each of the four *Mtb* strains with approximately 50–100 colony forming units (CFU) of each strain via the aerosol route using the Middlebrook Aerosol Exposure Chamber (Glas-Col, Terre Haute, IN). At days 0, 7, 14, 21, 28, 60, and 120 post-infection (p.i.), 5 mice per group were humanely euthanized for CFU determination in the lung and spleen, cytokine analysis and for lung pathology analysis.

### CFU count in lungs and spleens

Lungs and spleens from infected mice were excised, homogenized in sterile physiological saline (0.85% NaCl) and 10-fold serial dilutions prepared. With the exception of day zero, dilutions were plated on 7H11 agar plates and incubated for 21 days at 37°C until the enumeration of colonies. For day zero, whole lung preparations were plated. Data were expressed as Log_10_ CFU.

### Cytokine analysis

The Cytometric Bead Array (CBA, BD Biosciences) assay was used to determine cytokine concentrations after *Mtb* infection in the lung homogenates. Homogenates were clarified by centrifugation to remove cellular debris. In this assay, five bead populations coated with capture antibodies specific for Interleukin (IL) -2, IL-6, IL-10, Interferon (IFN)-γ and Tumor Necrosis Factor (TNF)-α were mixed with cell supernatants and detection was performed using the flow cytometer FACSCanto II (BD Biosciences) as described by the manufacturer. The final concentration of each cytokine was extrapolated from a standard curve using mouse cytokine proteins (from 0 to 5000 pg/mL) that define the minimum and maximum quantifiable levels. The limit of detection of each cytokine is stated at each result figure.

### Lung histology

During necropsy, the inferior lung lobe was inflated with 10% formalin/PBS and placed into 10% formalin/PBS. Following routine processing for paraffin-embedding, 5 μm sections were stained with hematoxylin and eosin (H&E) and assessed for degree of lung involvement by a veterinary pathologist blinded to the experimental groupings using a defined scoring system adapted from Dormans et al. [36]. The score was based on the total number of lesions, overall extent of changes, presence of peribronchiolitis, perivasculitis, alveolitis, “granuloma" formation and degree of necrosis. The scale ranges from zero (no apparent changes) to five (severe changes). Microphotographs were captured using a Nikon Eclipse 51E microscope equipped with a Nikon DS-Fi1 camera with a DS-U2 unit and NIS elements F software. Microphotographs are reproduced without manipulation other than cropping and adjustment of light intensity.

### Statistical analysis

A linear regression was built using the OD values from different concentrations of recombinant KatG to interpolate the KatG enzymatic activity in the cytosol and culture supernatants from the laboratory and clinical pairs. Statistical differences for peroxidase activity, Log_10_ CFUs and cytokine level comparisons were evaluated using an unpaired *t*-test or Mann-Whitney test depending on whether or not the data set had a normal distribution (defined by Kolmogorov-Smirnov test). Differences in lesion scores were analyzed using Kruskal-Wallis test and Dunn’s post-test. All statistical analyses were done in Graphpad Prism^®^ version 6.05.

## Results and Discussion

### *katG* N-terminus mutated *Mtb* INHr strains had strongly reduced peroxidase activity

**Fig 1:**
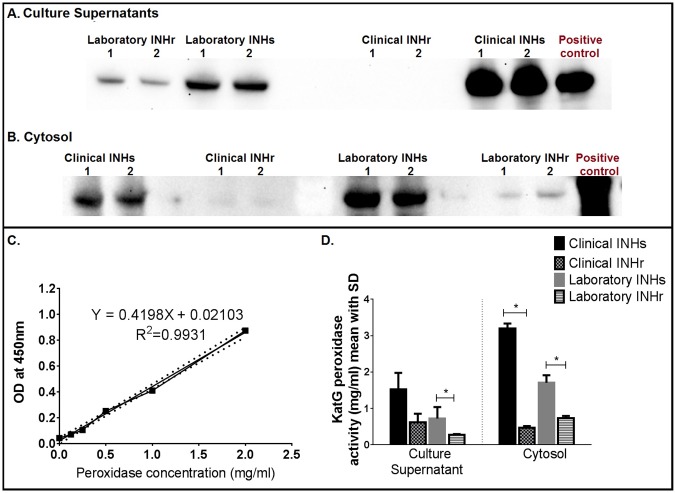
KatG levels and peroxidase activity in the clonal *Mtb* strains. A and B). Western blot using anti-KatG (from mouse clone IT-57) from culture supernatants and cytosol fractions of *Mtb* clonal strains cultured in GAS media respectively (1 and 2 represent the two culture replicates of each strain used). Recombinant KatG was used as a positive control. C). Standard curve for peroxidase activity using 3,3′,5,5′-Tetramethylbenzidine (TMB) as substrate and recombinant KatG. D). KatG peroxidase activity (derived from the standard curve, C) from laboratory and clinical *Mtb* pairs using recombinant KatG as reference. Non-paired *t*-test without assuming consistent standard deviation, *p<0.05.

Previous experiments suggest that *katG* mutations conferring INH resistance cause different levels of peroxidase activity in *Mtb* [32]. Therefore, we first wanted to estimate the levels of KatG and the peroxidase activity in our clinical and laboratory clonal pairs. *In vitro* cultures revealed lower levels of the protein and reduced peroxidase activity in the cytosol and culture supernatant of both INHr strains in comparison with equivalent protein quantities of their respective INHs strains (Fig 1). Catalase and peroxidase activities are both functions of KatG. They both involve the reduction of H_2_O_2_ and generation of water, however, KatG’s catalase and peroxidase catalytic functions are different as they require different hydrogen donors. Catalase reduces the H_2_O_2_ in a two-electron oxidation reaction using small organic substrates like ethanol with the generation of oxygen [37]. Peroxidase, instead, requires long-chain or aromatic compounds as hydrogen donors to complete its single-electron oxidation reaction [37]. In this study, we specifically measured the peroxidase activity since the aromatic compound 3,3′,5,5′-Tetramethylbenzidine (TMB) was used as the hydrogen donor.

Our results confirm that *katG* N-terminus single or double mutation (Val1Ala and Va1Ala plus the novel Glu3Val, observed in the laboratory and clinical INHr strain respectively) resulted in a significantly reduced intracellular and secreted levels of KatG and an associated lower peroxidase activity (p-value<0.05) (Fig 1). Under this condition, *Mtb* has an impaired ability to neutralize and overcome the highly oxidative environment in the phagosome and it could be rapidly cleared by macrophages and other phagocytes [12, 38]. Therefore, we conducted similar tests to determine whether or not there was a compensatory change in the levels of the AhpC protein in these *katG* mutants and the effect of this new phenotype in a mouse model of *Mtb* infection.

### Differential levels of AhpC levels were observed in N-terminus *katG* mutants

We evaluated if the reduced KatG levels observed in both INHr strains were concomitant with compensatory mutations in the promoter region of *ahpC* that could be associated with the up-regulation of AhpC in the laboratory INHr strain. Our sequencing results showed a wild type (WT) sequence for both the entire 105 bp *oxyR-ahpC* intergenic region and the *ahpC* gene in three biological replicates of the laboratory INHr strain (S1 and S2 Figs). A previous whole genomic sequencing (WGS) study demonstrated that both clinical INHs and INHr strains did not have any SNP for the *ahpC* gene or its promoter [31].

**Fig 2:**
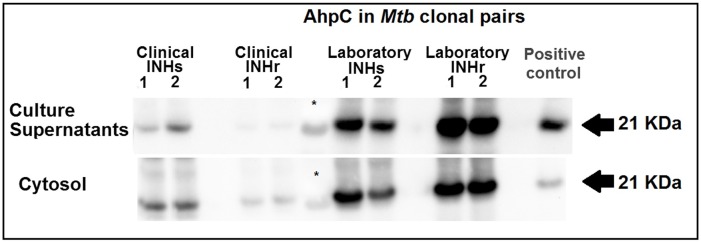
AhpC leves from soluble cellular fractions of *Mtb* cultures. Western blot using anti-AhpC_*Mtb*_ [17] from **c**ulture supernatants and cytosolic fraction of the four strains used in the study grown in GAS media (1 and 2 represent the two culture replicates of each strain used). CFP of H37Rv from BEI was used as a positive control. *indicates the lane for the ladder.

Western blot analysis of AhpC in soluble cell fractions on each of the *Mtb* strains demonstrated higher AhpC levels in both cytosol and secreted fraction in the laboratory INHr strain, compared to both its susceptible pair as well as both INHr and INHs clinical pair. On the contrary, AhpC levels were reduced in the clinical INHr strain compared to its INHs progenitor (Fig 2). This demonstrates that the reduced levels of KatG are being compensated by increased levels of AhpC without an associated SNP in this gene or its promoter in the laboratory INHr strain. Further, AhpC was more abundant in both laboratory strains when compared with the relative abundance of AhpC in the T genotype clinical pair (which was even lower in the clinical INHr strain). This suggests that the occurrence of different compensatory responses after acquisition of the *katG* mutation and subsequent resistant phenotype (INHr) may be dependent on the genetic lineage of the *Mtb* strain. Previous studies support this idea as they have demonstrated differences in the levels of some antigenic proteins and overall proteome among different *Mtb* genetic lineages [39, 40].

As mentioned, the AhpC levels in the clinical INHs strain are lower compared to the levels in the laboratory INHs strain (Fig 2). By contrast, the secreted levels of KatG in the clinical INHs strain are higher than in the laboratory INHs strain (Fig 1). This contributes to the idea that KatG and AhpC share enzymatic activity and substrates [17], since their levels appear to be complementary to each other in the INHs *Mtb* strains.

### In vivo growth of *Mtb* demonstrated reduced bacteria fitness in INHr strains

**Fig 3:**
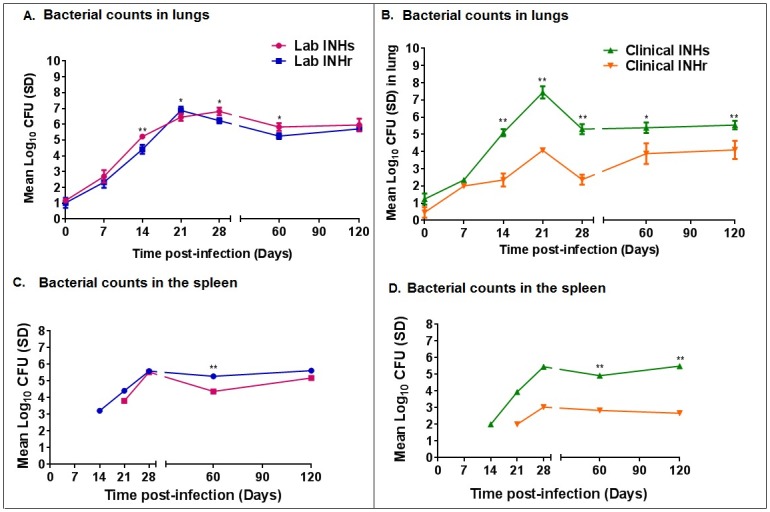
Comparison of bacterial growth in C57BL/6 mice after low dose aerosol infection. A) and C). Lung CFU count of mice infected with Laboratory and Clinical clonal strain pairs respectively. B) and D). Spleen CFU counts of Laboratory and Clinical clonal strain pairs respectively. Non-paired *t*-test without assuming consistent standard deviation (SD).*p<0.05, **p<0.001. Each time point represents the mean values for five mice infected with each of the four strains with the respective standard deviation shown in the error bars.

**Fig 4:**
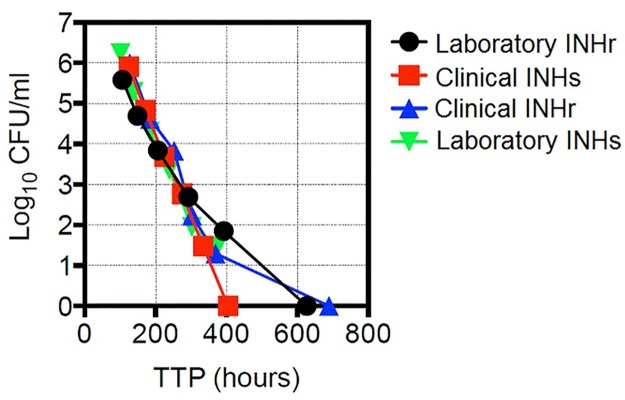
Comparison of bacterial growth rates in BACTEC media using MGIT^™^320. Time to positivity (TTP) of each strain was determined by seeding BACTEC tubes with 10-fold serial dilutions. TTP assessed in the MGIT^™^320.

The effect of the reduced KatG levels and enzymatic activity in the INHr strains was explored by looking at the differences in bacterial virulence compared to their correspondent INHs progenitor strains in a mouse model of infection. Significantly lower CFU counts were observed in the lungs and spleens of both clinical and laboratory INHr strains at several time points (p-value <0.05) (Fig 3). The reduction in bacterial counts was stronger in the clinical pair comparison, as bacterial counts were reduced up to approximately 3.0 log CFU on average in the spleen of mice infected with the clinical INHr strain, compared to its infecting INHs progenitor (Fig 3). The strongly reduced growth of the clinical INHr strain in the mouse tissues denote its impaired ability to infect and disseminate, showing a detrimental effect in its virulence and fitness. It is important to highlight that the bacterial growth of each strain was evaluated in BACTEC media using the MGIT^™^320 prior to the mouse infection. This confirmed the viability of all the strains and lack of significant differences in the bacterial growth and viability among them at the time of infection (Fig 4).

The decreased bacterial fitness observed particularly in the clinical INHr strain, has been demonstrated in the mycobacterial complex as an effect of some *katG* mutations using isogenic strains of *Mycobacterium bovis* and *Mtb* in the guinea pig and mouse models [38, 41, 42]. These studies were performed with a laboratory strain of *Mtb* with total deletion of the *katG* gene, a mutation rarely found in clinical settings [43]. In addition to this, there have been supportive reports describing differences in the CFU counts in mice infected with INHr clinical strains [14]. The study of the double mutant clinical INHr strain also confirmed the cumulative negative effect of the *katG* N-terminus mutations in bacterial fitness and adaptation to the host. It is important to emphasize that not every SNP combination in *katG* can cause a cumulative negative effect in the bacteria fitness and hence virulence. For instance, the Arg463Leu mutation in *katG* is not related with either INH resistance or virulence. This particular mutation instead has been used mostly for genotyping purposes [11].

### Differences in lung pathology at chronic stage of infection

**Fig 5:**
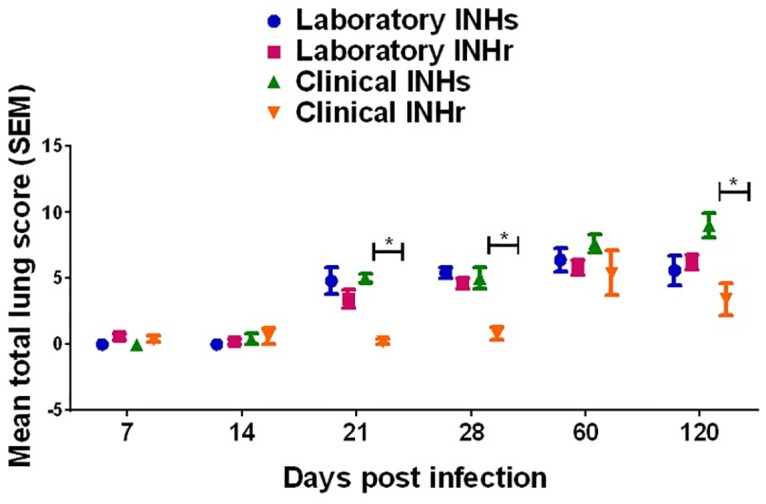
Lung pathology scores for C57BL/6 mice infected with clinical and laboratory clonal strains with different INH susceptibility profiles. Mean of the lung scores with standard error (SEM) for five infected mice with each strain differentiated by color. *p<0.05 with Kruskal-Wallis test and Dunn’s post-test.

To further evaluate how virulence was affected in the INHr strains, we compared the lesion in the lungs of the mice infected with the *Mtb* strains of our study. There were no statistical differences in the lung pathology scores at all the evaluated time points for the laboratory pair comparison. Pathologic findings in animals infected with both laboratory INHs and INHr strains included peri-bronchiolitis, peri-vasculitis and alveolitis from day 21 p.i. onwards, with marked granulomatous inflammation from day 60 p.i. onwards. In contrast, the lung pathology scores of the mice infected with the clinical INHr strain at days 21, 28 and 120 p.i. were significantly lower than scores for the clinical INHs strain (Fig 5). Mice infected with the clinical INHs strain also showed signs of inflammation from day 21 p.i., while for the clinical INHr infected animals, inflammatory lesions were rarely observed until day 60 p.i.

A prior study using the clinical strains (labeled as T21 and T27) suggested no significant differences in lung pathology during the first 90 days of infection between the INHr and INHs strains [44]. In our study, we were able to fully evaluate the pathology of these strains with a detailed scoring system; from this, we built upon the previous assessment and observed clear differences in the lung pathology during the course of infection, with the greatest difference observed at the latest time point (day 120 post-infection) (Fig 5 and S3 Fig).

### Lower induction of proinflammatory cytokines after infection with INHr *Mtb*

**Fig 6:**
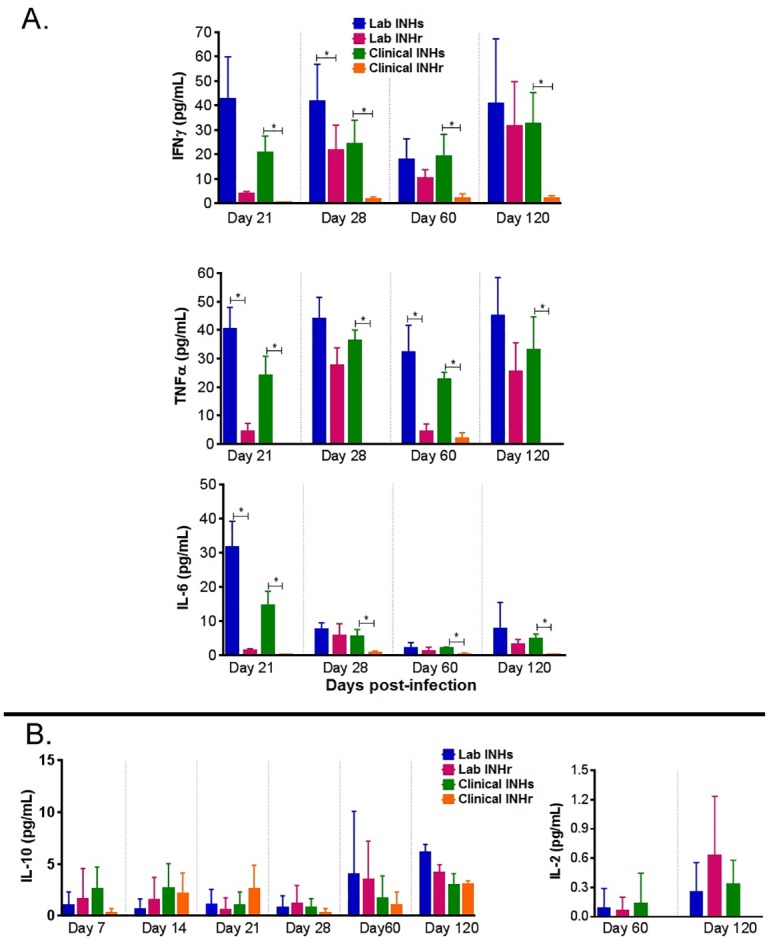
Dynamics and comparison of cytokine levels in mouse lung homogenates. A. Comparison of pro-inflammatory cytokines levels throughout the infection with pairs of clinical and laboratory clonal *Mtb* strains (t-test, *p<0.05). Limit of detection (LD) for IFN-γ: 0.5pg/mL, TNF-α: 0.9 pg/mL, and IL-6: 1.4 pg/mL.) Pair comparison between mice infected B). IL-10 (LD: 16.8 pg/mL) and IL-2 (LD: 0.1 pg/mL) levels in mice infected with laboratory and clinical *Mtb* pairs. Bars represent the mean values of cytokine concentration for five mice and the error bars represent the standard deviation.

To determine the immune response generated against these less virulent INHr strains, we performed an evaluation of cytokines in the mouse lung tissue. The dynamics of the proinflammatory cytokines IFN-γ, TNF-α, and IL-6 levels as well as IL-10 and IL-2 in both INHr strains compared to their INHs progenitor were evaluated throughout the study (Fig 6A). Again, the significant reduction of the three proinflammatory cytokines was observed particularly in the animals infected with the clinical INHr strain (from day 21 p.i. onward) (Fig 6A). On the other hand, although not significant, there was a slight increase of IL-10 in the laboratory INHr strain at the initial time points of infection (day 7 and 14 p.i.) and for the clinical INHr strain only at day 21 p.i) (Fig 6B). In addition, there were not significant differences for IL-2 in the clonal pair comparison, although the clinical INHr strain did not have detectable levels for this cytokine in most of the evaluated time points (data shown only for day 60 and 120 p.i.). Substantial reduction in the levels of the proinflammatory cytokines IFN-γ and TNF-α, together with the slightly augmented levels of the anti-inflammatory cytokine IL-10 in mice infected with the clinical INHr strain (Fig 6) are in line with the deficient granulomatous reaction with lower cell infiltration observed in the lungs of these mice (S3 Fig).

Mice infected with the clinical INHr strain controlled the bacterial load and exhibited decreased pathology, despite the apparent lack of macrophage activation (lower induction of proinflammatory cytokines). Two events are hypothesized to explain this observation: first, the reduced levels of the catalase-peroxidase KatG and the hydroperoxidase AhpC (reduction of a bacterial virulence factor and anti-redox stress response proteins) allowed a more rapid elimination of the infecting bacteria. Secondly, the minimal induction of a Th1 response was sufficient to clear the attenuated bacteria from the lung, generating less lung tissue damage (S3 Fig) and reduced bacterial dissemination (Fig 1D). While macrophage activation and phagocytosis are key features traditionally required to control *Mtb* growth during infection [20], other innate immune factors and cells, such as invariant natural killer T cells, γδT cells or Mucosa-associated invariant T (MAIT) cells [45, 46] could potentially play an important role in the clearance of the less virulent INHr strain.

## Conclusions

To our knowledge, this is the first time that a virulence study of INHr *Mtb* strains integrated a biochemical and immunopathological evaluation using clinical and laboratory derived clonal *Mtb* strains. First, we performed an *in vitro* analysis of their KatG and AhpC protein levels, peroxidase activity and sequence analysis of the respective genes. Second, the CFU count, lung cytokine profile and histopathology were assessed after infecting mice with the characterized clonal *Mtb* pairs. The two *katG* mutations evaluated here have been found in a low frequency in clinical settings (one of them is novel), which suggests a lower fitness for these INHr strains [10, 13]. However, the reduced KatG levels and peroxidase activity alone were not enough to explain the significantly reduced virulence and pathology scores observed mainly for the clinical INHr strain. Therefore, it is possible that compensating events occurring in the laboratory INHr strain resulted in just a subtle reduction in virulence. Our findings support the previously discussed role of AhpC as a compensatory mechanism of *katG* mutant strains, restoring their anti-oxidative stress capacity [17, 18, 47]. The fact that the AhpC levels were different among clonal strains without a related genetic defect in the *ahpC* gene or its promoter supports the idea that phenotypic diversity is not always a direct consequence of genetic variation [48].

Overall, we provided evidence of the complex nature of INH resistance, from its origins (which includes multiple mutations [10]) to the resulting bacterial phenotype. Specifically, our study supports that bacterial virulence, pathogenicity and fitness depend not only on the specific SNP that drives the INHr event, but also on the genetic background or bacterial lineage itself, which will ultimately determine how the bacterium successfully adapts to the new phenotype. Therefore, our findings emphasize the importance of studying clonal *Mtb* strains in the description of a biological event in order to get a more accurate description and minimizing possible confounding factors. Further physiological rearrangements could occur in the INHr strains that may include a variation in its metabolic profile which can be explored in a more comprehensive biochemical analysis. Moreover, it is also important to evaluate the early anti-TB innate immune response that probably contributes to the efficient clearance of some INHr strains.

## Supporting Information

S1 Fig Sequence alignment results for the *oxyR-ahpC* region in the laboratory clonal pair. Alignment performed with the forward *oxy-ahpC* primer.
(TIF)

S2 Fig Sequence alignment results for the *ahpC* gene in the laboratory clonal pair. Alignment performed with the forward *ahpC* primer.
(TIF)

S3 Fig Histopathology for C57BL/6 mouse lungs infected with clinical strains with significant differences. Four examples of the spectrum of lesions and corresponding scores are depicted. H&E stained sections at 20x and 100x original magnifications.
(TIF)

S1 Table Primer sequence used for PCR-Sanger sequencing of *ahpC* and its promoter. (DOCX)
