# Supplementary figures and images for: Virulence of Mycobacterium tuberculosis after Acquisition of Isoniazid Resistance: Individual Nature of katG Mutants and the Possible Role of AhpC
Source: PLoS One. 2016 Nov 28;11(11):e0166807. doi: 10.1371/journal.pone.0166807 (PMC5125630; doi:10.1371/journal.pone.0166807)

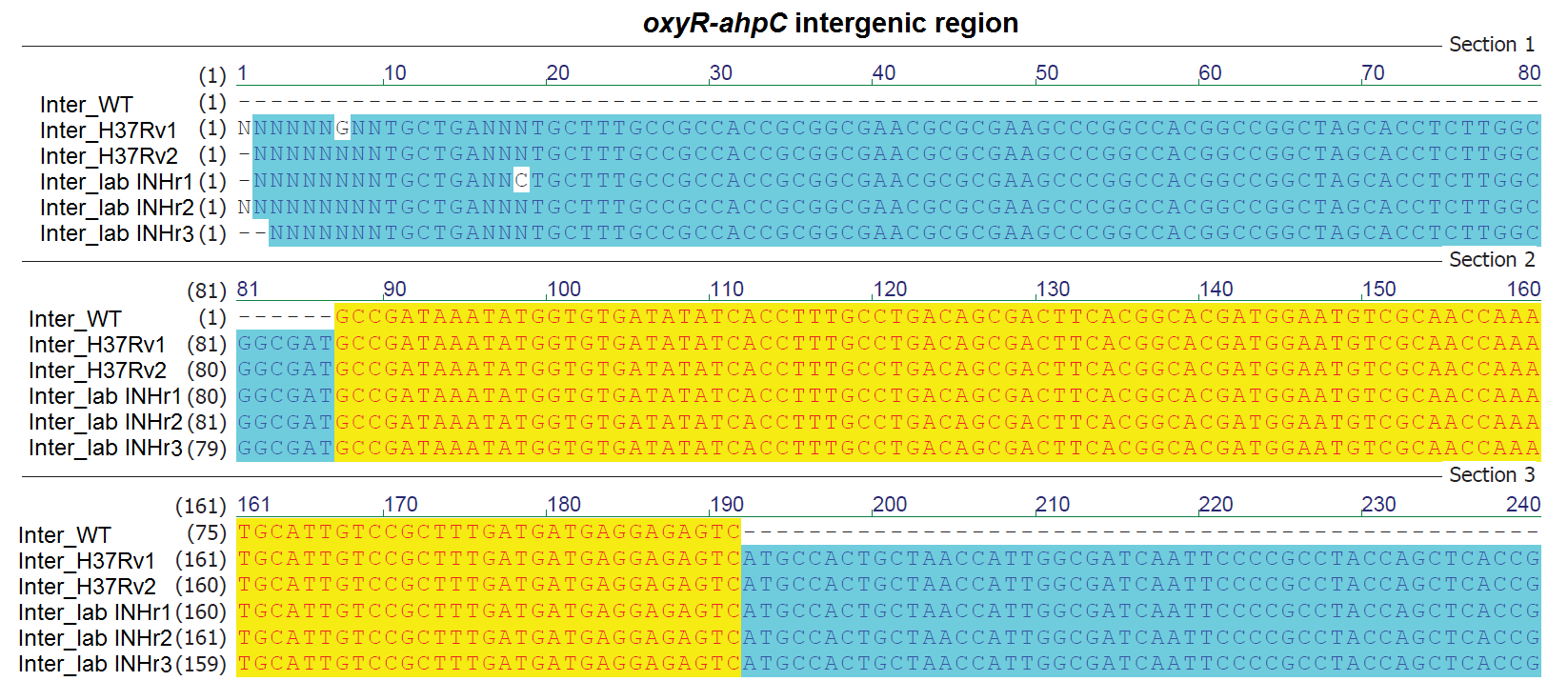

Supplement: S1 Fig — Alignment performed with the forward oxy-ahpC primer. (TIF) [file pone.0166807.s001.tif]

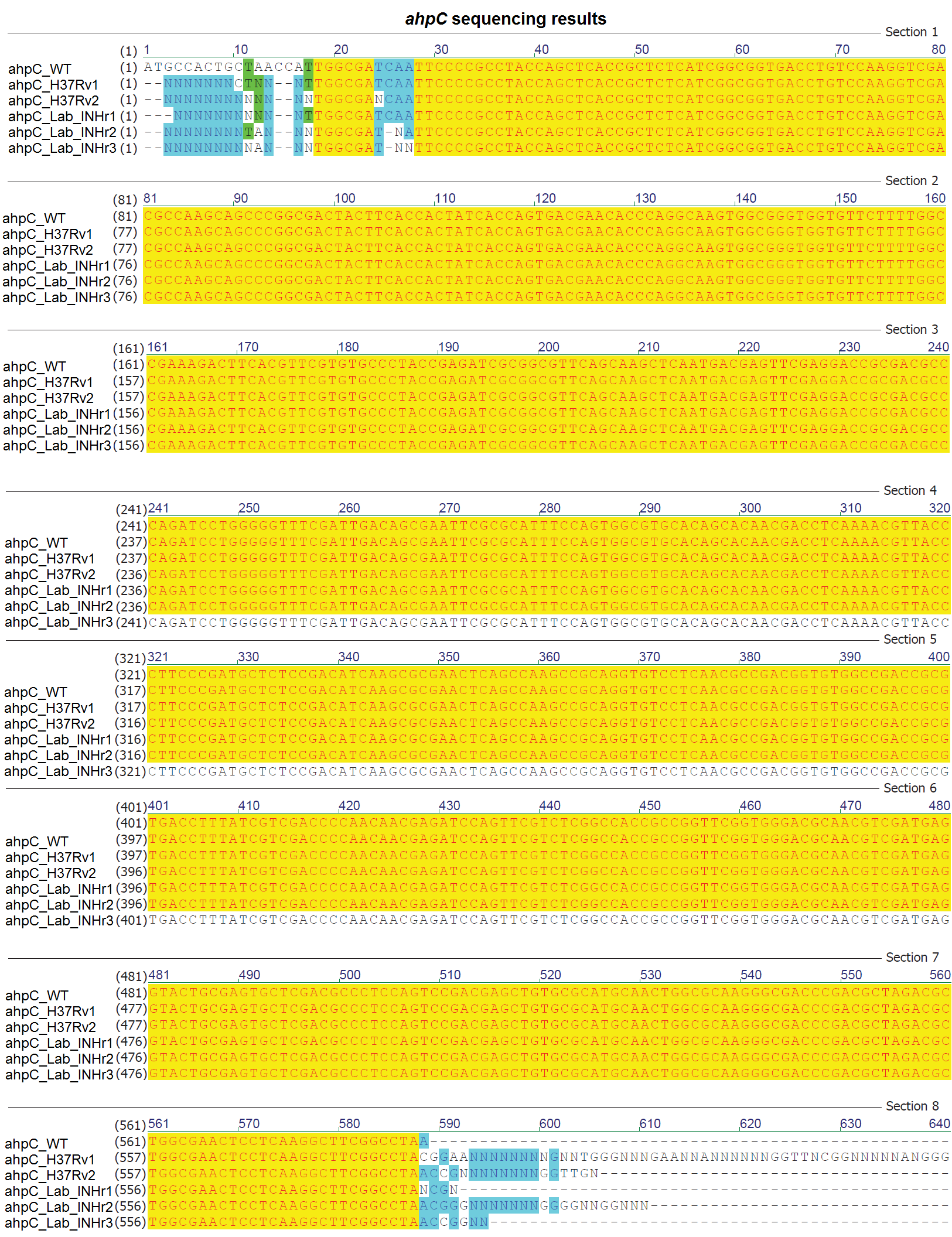

Supplement: S2 Fig — Alignment performed with the forward ahpC primer. (TIF) [file pone.0166807.s002.tif]

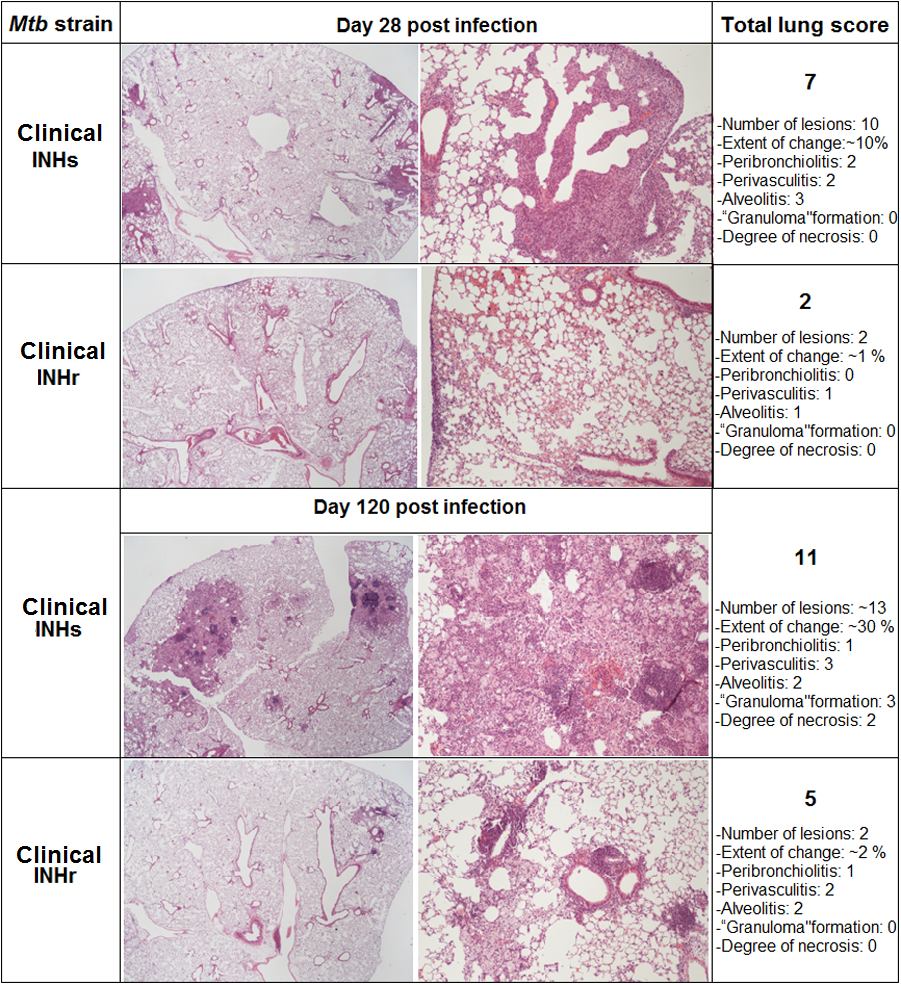

Supplement: S3 Fig — Four examples of the spectrum of lesions and corresponding scores are depicted. H&E stained sections at 20x and 100x original magnifications. (TIF) [file pone.0166807.s003.tif]
