# Supplementary material for: Virulence of Mycobacterium tuberculosis after Acquisition of Isoniazid Resistance: Individual Nature of katG Mutants and the Possible Role of AhpC
Source: PLoS One. 2016 Nov 28;11(11):e0166807. doi: 10.1371/journal.pone.0166807 (PMC5125630; doi:10.1371/journal.pone.0166807)
Supplement: S1 Table — (DOCX) [file pone.0166807.s004.docx]

**Supplementary table 1. Primer sequence used for PCR-Sagen sequencing of ahpC and its promoter.**

| Sequence to amplify | Primer |
| --- | --- |
| Intergenic region  *oxyR-ahpC* | Forward: 5’ CATTGTCCGCTTTGATGATGAG 3’  Reverse: 5’ CAACCAGATCCCGGTTAGG 3’ |
| *ahpC* | Forward: 5’- AACGTCGACTGGCTCATATC -3’  Reverse: 5’-TGGTGATAGTGGTGAAGTAGTC- 3‘ |
